# Effect of labor dance on induction duration, first-stage labor, and maternal anxiety in pregnant women with term premature rupture of membranes: A randomized controlled trial

**DOI:** 10.18332/ejm/219323

**Published:** 2026-03-31

**Authors:** Jinxuan Zheng, Biyun Wang, Dongmei Duan, Yingfang Wu, Luhuan Yang

**Affiliations:** 1 Department of Nursing, Xiaolan Clinical Institute of Shantou University Medical College, Zhongshan, China; 2 Department of Obstetrics, Dongguan City Maternal and Child Health Hospital, Dongguan, China; 3 Department of Community Health, Faculty of Medicine and Health Sciences, University of Putra Malaysia, Serdang, Malaysia

**Keywords:** induction of labor, oxytocin, term, premature rupture of membranes, labor dance

## Abstract

**INTRODUCTION:**

Labor dance is an emerging non-pharmacological intervention for labor support, though current evidence remains limited, especially among pregnant women with term premature rupture of membranes (TPROM). This study aims to evaluate the effect of labor dance on the duration of labor induction and the first stage of labor, as well as on maternal and neonatal outcomes and maternal anxiety.

**METHODS:**

A randomized controlled trial was conducted at a tertiary maternal and child healthcare hospital in southern China from 20 November 2023 to 31 October 2024. A total of 102 eligible full-term pregnant women with premature rupture of membranes (PROM), aged 18–35 years, and primiparous with adequate health literacy and capacity for informed consent, were randomly allocated in a 1:1 ratio to either the intervention or control group. The control group received oxytocin-induction management with routine care, ambulation encouragement. The intervention group received the same care plus labor-dance sessions (8–10 minutes per sequence, at least every 30 minutes) from 0 to 5–6 cm cervical dilation. The primary outcomes measured were the duration of labor induction. Secondary outcomes included the length of the first stage of labor, maternal anxiety levels, postpartum hemorrhage within 24 hours, delivery mode, and the incidence of maternal and neonatal infections.

**RESULTS:**

Compared with the control group, the intervention group had a shorter induction time (406.20 ± 177.53 vs 495.98 ± 188.22 min), with a mean difference of 89.78 minutes (95% CI: 13.99–165.57, p=0.021). Among participants who delivered vaginally, there was a shorter first stage (429.14 ± 141.39 vs 509.28 ± 167.54 min), with a mean difference of 80.14 min (95% CI: 12.59–147.68, p=0.021). Post-intervention anxiety scores were lower in the intervention group (median: 4 vs 5; p<0.05). Chorioamnionitis occurred less frequently (13.0% vs 30.4%; p<0.05). Total labor duration, 24-h postpartum hemorrhage, overall delivery mode, and neonatal complications (asphyxia/pneumonia) did not differ significantly between groups (all p>0.05).

**CONCLUSIONS:**

In pregnant women with TPROM undergoing oxytocin induction, labor dance – a low-risk adjunct – was associated with shorter induction and first-stage labor, lower anxiety, and maybe reduced chorioamnionitis, without increases in postpartum hemorrhage or neonatal complications.

**CLINICAL TRIAL REGISTRATION:**

The study is registered on the official website of the Chinese Clinical Trails Registry

**IDENTIFIER:**

ChiCTR2300073552

## INTRODUCTION

Term premature rupture of membranes (TPROM) refers to spontaneous rupture of fetal membranes at or beyond 37 weeks of gestation prior to the onset of labor^1^. TPROM presents a common but clinically challenging obstetric condition because, once the membranes rupture, prolongation of the interval to delivery may increase the risk of maternal and neonatal complications^1,2^. In particular, the risk of infectious morbidity for both the mother and neonate rises progressively with the duration between TPROM and delivery^3^. Therefore, timely labor management is essential for women with TPROM in order to reduce infection-related risks and optimize perinatal outcomes.

Induction of labor (IOL), defined as the artificial initiation of uterine contractions before the spontaneous onset of labor, is a standard obstetric practice recommended for the management of TPROM^1,3^. In current clinical practice, pharmacological methods, particularly oxytocin and prostaglandins, are commonly used to initiate labor in this setting^4^. However, although these approaches are widely used, they are not without limitations. Previous studies have reported failure rates of approximately 15% to 20%, which may ultimately necessitate operative delivery or emergency cesarean section^5^. In addition, repeated oxytocin exposure has been associated with adverse effects such as excessively strong uterine contractions, fetal distress, and, in rare cases, uterine rupture. These challenges may also contribute to increased maternal anxiety and distress during labor^6,7^. Taken together, these limitations suggest that supportive adjunctive strategies may be needed to complement pharmacological induction and improve both labor progress and the maternal childbirth experience.

In this context, nonpharmacological interventions such as labor dance have attracted increasing attention. Labor dance is a supportive intervention involving upright positioning, rhythmic movement, and pelvic motion during labor^8^. Previous studies suggest that labor dance may help reduce maternal discomfort, alleviate childbirth-related anxiety or fear, and support favorable labor progress^9-11^. However, the available evidence remains limited, particularly among women undergoing oxytocin-induced labor after TPROM. Therefore, this study aimed to evaluate the effects of labor dance as an adjunct to oxytocin-induced labor in primigravida women with TPROM, with a particular focus on induction-to-delivery duration, the length of the first stage of labor, maternal anxiety, and selected maternal and neonatal outcomes. By examining this intervention in a specific induced-labor context, we sought to provide preliminary evidence to inform supportive obstetric care.

## METHODS

### Design and settings

A randomized controlled trial was conducted at the delivery center of a tertiary maternal and child health hospital in Southern China from 20 November 2023 to 31 October 2024 (A CONSORT checklist is provided in the Supplementary file).

The center is a specialized unit with 10 single-occupancy rooms that cares for more than 6500 pregnant women annually, approximately 2% of whom present with term premature rupture of membranes (TPROM).

### Participants

Primigravida women diagnosed with TPROM at 37–42 weeks of gestation who were scheduled for oxytocin induction were screened for eligibility^12^.

Inclusion criteria included primigravida, singleton pregnancy, age 18–35 years, confirmed TPROM at 37–42 weeks of gestation prior to the onset of labor, stable maternal and fetal condition, cephalic presentation, modified Bishop score ≥7 ^13^, estimated fetal weight 2500–4000 g, normal fetal heart rate, indication for induced labor 4, and ability to communicate effectively.

Exclusion criteria included pregnancy complications such as oligohydramnios, polyhydramnios, placenta previa, pregnancy-induced hypertension, preeclampsia, fetal malposition, abnormal fetal head engagement, or fetal distress.

Attrition criteria within our RCT included: 1) emergency cesarean section during the intervention; 2) temporary discontinuation of intravenous oxytocin during IOL because of clinical concerns, such as fetal heart rate deceleration; 3) use of other non-pharmacological interventions, such as acupressure or hydrotherapy during IOL; and 4) receipt of epidural or other analgesia.

### Sample size

To the best of our knowledge, the primary outcome of this study – labor induction duration – has not been reported in the peer-reviewed literature. As a result, given the absence of publicly available data on critical parameters such as effect size and variability in existing literature, traditional sample size estimation methods are not feasible. Therefore, this study is designed as an exploratory and feasibility trial. The sample size was determined based on the projected number of eligible participants anticipated for enrollment at our institution within a year, with a target of enrolling 120 term pregnant women with premature rupture of membranes.

### Randomization

Randomization used computer-generated permuted blocks (size 4) with 1:1 allocation, concealed via sequentially numbered, opaque, sealed envelopes opened only after consent. Midwives and participants were unmasked; outcome assessors and statisticians were blinded until database lock.

### Interventions

Delivery-room conditions (temperature, lighting, audio equipment, and continuous fetal heart rate monitoring) were standardized for both groups. Intravenous oxytocin was initiated using either 10 IU in 1000 mL or 5 IU in 500 mL isotonic saline, starting at 3.3 mIU/min with 3.3 mIU/min increments every 20 min until regular uterine activity (3–5 contractions per 10 min) was achieved^14^. When cervical dilation reached 6 cm with regular contractions, the infusion was discontinued^15^. If a primiparous woman had regular contractions for ≥12 h but remained in the latent phase, oxytocin was stopped and delivery mode was converted to cesarean section per protocol^15^.

*Control group*

Participants in the control group received routine nursing care during oxytocin induction, including counseling on a high-protein diet, rest in the left lateral and reverse Trendelenburg positions, continuous fetal monitoring, and vaginal examinations. In addition, walking was encouraged as tolerated, unless contraindicated, as part of routine obstetric care^16^. During labor, vaginal examination frequency followed clinical protocols: before dilation, based on pain and contractions; during the first stage, every 4 hours (latent) and 2 hours (active), with additional exams as indicated by maternal symptoms.

*Intervention group*

In addition to the routine care, participants performed labor dance during effective regular contractions, contingent on maternal–fetal wellbeing. Sessions took place in private rooms and were led by three midwives certified in the International Childbirth Dance Program. Choreography followed Gönenç et al.^11^ and comprised: 1) pelvic lateral oscillation (side-to-side pelvic movement in the horizontal plane), 2) circular hip rotations (pelvic rotation around the hip axis in the horizontal plane), and 3) a standing pelvic tilt. Participants maintained an upright torso with knees flexed at about 15° and feet shoulder-width apart; for the standing tilt, they leaned forward at about 15°. During the upright tilt, arms rested around the midwife’s shoulders for support while the researcher applied a gentle lower back touch massage^17^. Music in 3/4 time was used to facilitate rhythm and flow^18^.

*Labor dance duration and frequency*

Labor dance was performed from the start of oxytocin induction and repeated approximately every 30 min until cervical dilation reached 5–6 cm. Each sequence followed the order: lateral oscillation → circular hip rotations → standing pelvic tilt; sequences used eight counts per movement and lasted 5–6 min, with the standing pelvic tilt held for 3–4 min^11^.

To ensure intervention fidelity, all midwives underwent standardized training and delivered the intervention according to a unified protocol developed with reference to the study by Gönenç et al.^11^. Weekly calibration meetings were also conducted to maintain consistency in intervention delivery across midwives.

### Data collection

The variable data in this study were obtained from the hospital’s electronic medical record system and patient inquiries. Variables such as age, gestational weeks, pre-pregnancy body mass index, antibiotic use, duration of membrane rupture, cervical score, and education level were all extracted from the hospital’s electronic medical record system. The newborn’s weight was obtained from B-ultrasound examinations. Additionally, work status and income level were obtained through inquiries. In addition, cervical favorability was assessed using the modified Bishop score, and only participants with a score of ≥7, indicating a cervix suitable for oxytocin induction without prior ripening, were included in the study^13^.

### Outcomes

*Primary outcome*

The prespecified primary outcome was duration of IOL. The IOL was calculated in minutes as the interval from initiation of intravenous oxytocin to protocol-directed discontinuation.

*Secondary outcomes*

Labor duration

First-stage duration and total labor duration were measured prospectively using time-stamped entries from the partograph and the electronic medical record. The first-stage duration was calculated in minutes from the first documented onset of regular uterine contractions to complete cervical dilation (10 cm). The total labor duration was calculated in minutes from the onset of regular contractions to the documented time of placental delivery. These labor duration outcomes were assessed only among women who achieved vaginal delivery.

Anxiety

Anxiety was assessed with the Visual Analog Scale for Anxiety (VAS-A), a 10 cm horizontal line anchored at 0 (‘no anxiety’) and 10 (‘maximum anxiety’), scored to the nearest cm^19,20^. Consistent with prior literature, a value of ≥5 cm was treated as a clinically meaningful threshold for anxiety^21^. VAS-A scores were assessed at two predefined time points: 1) baseline – defined as the time immediately following randomization and initiation of oxytocin infusion; and 2) post–induced labor – defined as the time immediately following completion of the induced labor procedure.

Maternal–fetal outcomes

Maternal-fetal outcomes included 24-h postpartum hemorrhage, mode of delivery, and infection events, each measured from the medical record using predefined operational rules. Postpartum hemorrhage was recorded in mL and tallied cumulatively from delivery to 24 hours postpartum. Mode of delivery was abstracted from the delivery note and coded as normal vaginal delivery (NVD), assisted vaginal delivery (AVD), or cesarean section (CS). Infection events were counted only when a clinician-entered diagnosis appeared in the chart during the index hospitalization: maternal infection as chorioamnionitis and/or acute endometritis, and neonatal complications as neonatal asphyxia and/or neonatal pneumonia.

### Safety monitoring

To safeguard maternal and fetal well-being, all participants underwent continuous electronic fetal monitoring throughout labor induction and active labor. Maternal condition was closely monitored throughout the intervention through regular verbal checks and direct observation. The intervention was immediately suspended if any clinically significant abnormality occurred, including fetal distress, uterine hyperstimulation, abnormal fetal heart rate patterns, or maternal symptoms such as dizziness, shortness of breath, or marked discomfort. Subsequent clinical actions were guided by standard obstetric protocols. If adverse events met the prespecified study withdrawal criteria, the participant was withdrawn from the intervention according to the protocol.

### Ethics

This trial was registered with the Chinese Clinical Trial Registry (ChiCTR2300073552) and obtained ethical approval from the Ethics Committee of Dongguan City Maternal and Child Health Hospital (No.202351) on 25 June 2023. All participants provided written informed consent after receiving information about the study objectives and procedures, and they were free to withdraw at any stage without consequences.

### Statistical analysis

Raw data were collected using Microsoft Excel and analyzed with SPSS 21.0. Categorical data were summarized using frequencies and percentages. Continuous variables were tested for normality using the Shapiro-Wilk test. Group differences were assessed using the chi-squared test. For normally distributed continuous data, data were presented as mean ± standard deviation (SD) and compared using the independent-samples t-test. Non-normally distributed continuous variables were described using the median and interquartile range (IQR) and compared using the Mann–Whitney U test or Wilcoxon rank-sum test, as appropriate. All statistical tests were two-tailed, and statistical significance was set at p<0.05.

The primary analysis was conducted on the per-protocol population. Because 10 randomized participants had no post-randomization data for the protocol-defined primary outcomes, a strict intention-to-treat analysis based on observed outcome data was not performed.

## RESULTS

Of 120 women assessed for eligibility, 18 were excluded (twins, n=2; declined to participate, n=11; age >35 years, n=2; gestational hypertension, n=2; oligohydramnios, n=1). The remaining 102 primigravida women provided consent and were randomized in a 1:1 ratio to the intervention group (n=51) or the control group (n=51).

**Figure 1:**
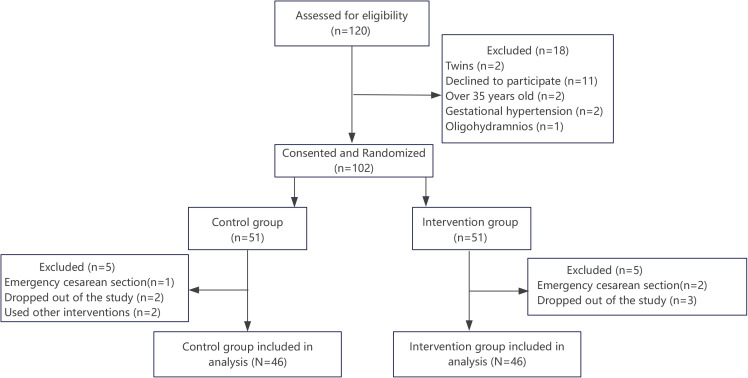
Flow chart of participant selection

During follow-up, five participants in each group were excluded from analysis. In the intervention group, three withdrew for personal reasons, and two underwent emergency cesarean section because of fetal distress during induction. In the control group, two withdrew for personal reasons, one underwent emergency cesarean section for fetal distress, and two received non-protocol nonpharmacological measures (birthing ball or hydrotherapy). No participant in the labor dance group required discontinuation of the intervention because of fatigue. Consequently, 92 participants were included in the final analysis (46 per group) (Figure 1).

To explore the potential impact of attrition, baseline characteristics of the 92 participants included in the final analysis were compared with those of the 10 excluded participants. No statistically significant differences were observed between the two groups in age, gestational age (weeks), pre-pregnancy body mass index (BMI), cervical score, or antibiotic administration (all p>0.05). The number of exclusions was the same in both study groups, with five participants excluded from each group.

### Sociodemographic characteristics

Of the 92 participants included in the analysis, 53.26% were aged 18–30 years, and 79.35% were between 37 and 39 weeks of gestation. No significant differences were observed between the intervention and control groups regarding age or gestational weeks (p>0.05). Similarly, there were no statistically significant differences in education level, employment status, income level, pre-pregnancy body mass index, newborn weight, or duration of ruptured membranes between the two groups (p>0.05). Detailed demographic characteristics are presented in Table 1.

**Table 1:** Baseline characteristics of participants before intervention at the delivery center of a tertiary maternal and child health hospital in Southern China, 20 November 2023 – 31 October 2024 (N=92)

*Variables*	*Control group* *(N=46)* *n (%)*	*Intervention* *Group (N=46)* *n (%)*	*Χ^2^/t*	*p*
**Age** (years)			0.393	0.531^†^
22–30	26 (56.52)	23 (50.00)		
31–34	20 (43.48)	23 (50.00)		
**Education level**			0.090	0.765^†^
High school	6 (13.04)	7 (15.22)		
University	40 (86.96)	39 (84.78)		
**Work status**			0.205	0.650^†^
Work	31 (67.39)	33 (71.74)		
No work	15 (32.61)	13 (28.26)		
**Income level**			0.073	0.964^†^
Low	9 (19.57)	10 (21.74)		
Middle	25 (54.35)	24 (52.17)		
High	12 (26.09)	12 (26.09)		
**Pre-pregnancy BMI** (kg/m²)			0.949	0.622^†^
<18.5	4 (8.70)	7 (15.22)		
18.5–24.9	36 (78.26)	33 (71.74)		
≥25	6 (13.04)	6 (13.04)		
**Gestational weeks**			0.066	0.797^†^
37–39	36 (78.26)	37 (80.43)		
40–41	10 (21.74)	9 (19.57)		
**Neonatal weight** (g)			0.555	0.758^†^
<2500	5 (10.87)	6 (13.04)		
2500–4000	36 (78.26)	33 (71.74)		
>4000	5 (10.87)	7 (15.22)		
**Administration of antibiotics**			0.103	0.748^†^
No	40 (86.95)	41 (89.13)		
Yes	6 (13.04)	5 (10.87)		
**Cervical score**			0.472	0.492^†^
7–8	34 (73.91)	31 (80.43)		
9–10	12 (26.87)	15 (32.61)		
**Duration of membrane rupture** (h)			0.085	0.933
Mean (SD)	451.39 (187.06)	454.70 (187.30)		

† χ² test.

### The average number of sequences

In the intervention group, labor dance was initiated at the start of oxytocin infusion and was repeated until cervical dilation reached 5–6 cm. Therefore, the total number of completed dance sequences per participant varied according to individual labor progression and induction duration. The median duration of birthing dance exposure was 73.33 min (interquartile range, IQR: 50.21–100.00), and participants completed a median of 15 sequences (IQR: 10–20). All participants completed at least one full sequence.

### Effect of labor dance on labor duration

For all participants included in the analysis (N=92), the induction time in the intervention group was significantly shorter than in the control group (406.20 ± 177.53 vs 495.98 ± 188.22 min), with a mean difference of 89.78 min (95% CI: 13.99–165.57, p=0.021). Among participants, 83 women achieved vaginal delivery following labor induction, including 43 in the intervention group and 40 in the control group. Among the 83 participants, the first stage of labor was notably shorter in the intervention group (429.14 ± 141.39 min) than in the control group (509.28 ± 167.54 min, p<0.05). Similarly, the total duration of labor was shorter in the intervention group (475.95 ± 146.66 min) compared to the control group (542.55 ± 167.76 min), but this difference did not reach statistical significance (p>0.05). Detailed data are presented in Table 2.

**Table 2:** Comparison of duration of labor between two groups of women who achieved vaginal delivery after induced labor following intervention (N=83)

*Variables*	*Intervention* *group* *(N=43)* *Mean (SD)*	*Control group* *(N=40)* *Mean (SD)*	*MD*	*95% CI*	*t*	*p*
Duration of the first stage of labor (min)	429.14 (141.39)	509.28 (167.54)	80.14	12.59–147.68	2.361	0.021*
Total duration of labor (min)	475.95 (146.66)	542.55 (167.76)	66.60	-2.09–135.687	1.929	0.057

MD: mean difference. t: Student’s t-test.

* p<0.05 significance level.

### Effect of labor dance on anxiety

Prior to the intervention, the median anxiety scores were similar between the intervention and control groups, with no statistically significant difference observed (6 points vs 5 points, p>0.05). Following the intervention, anxiety levels decreased in both groups. However, the median anxiety score in the intervention group dropped significantly lower than that observed in the control group (p<0.05). Detailed data are presented in Table 3.

**Table 3:** Comparison of changes in VAS-A scores for anxiety between the two groups before and after the intervention (N=92)

*Variable*	*Control group (N=46)*		*Intervention group (N=46)*		*Test statistics* *between groups*
	*Median (IQR)*	*Test statistics* *within groups*	*Median (IQR)*	*Test statistics* *within groups*	
Pre-test anxiety	6.0 (5.0–7.0)	W*=3.999 p<0.001	5.0 (4.0–7.0)	W*=5.800 p<0.001	U=0.782 p=0.434
Post-test anxiety	5.0 (4.0–6.0)		4.0 (3.0–5.0)		U=2.925 p=0.003*

IQR: interquartile range. U: Mann Whitney U test. W*: Wilcoxon test.

* p<0.05 significance level.

### Effect of labor dance on maternal and fetal outcomes

We found no significant difference in median postpartum blood loss between the intervention and control groups (300 vs 400 mL, p>0.05).

Regarding mode of delivery, NVD occurred in 42/46 (91.30%) participants in the intervention group and 37/46 (80.43%) in the control group, with no statistically significant between-group difference (p>0.05). Overall vaginal delivery (NVD + AVD) occurred in 43/46 (93.48%) participants in the intervention group and 40/46 (86.96%) in the control group, also without a statistically significant difference (χ²=0.49, p>0.05).

Chorioamnionitis occurred less frequently in the intervention group than in the control group [6/46 (13.04%) vs 14/46 (30.43%), p<0.05]. The corresponding odds ratio was 0.343 (95% CI: 0.118–0.993), suggesting lower odds in the intervention group.

No significant between-group differences were observed in acute endometritis [5/46 (10.87%) vs 6/46 (13.04%)], neonatal asphyxia [1/46 (2.17%) vs 2/46 (4.35%)], or neonatal pneumonia [2/46 (4.35%) vs 2/46 (4.35%)] (all p>0.05) (Table 4).

**Table 4:** Comparison of maternal and fetal outcomes between two groups after the intervention (N=92)

*Variable*	*Control group* *(N=46)* *n (%)*	*Intervention* *group (N=46)* *n (%)*	*Total* *(N=92)* *n (%)*	*χ^2^*	*p*
**Delivery mode**				2.383	0.304
NVD	37 (80.40)	42 (91.30)	79 (85.90)		
AVD	3 (6.50)	1 (2.20)	4 (4.30)		
CS	6 (13.0)	3 (6.50)	9 (9.80)		
**Chorioamnionitis**				4.089	0.043*
No	32 (69.57)	40 (86.96)	72 (78.26)		
Yes	14 (30.43)	6 (13.04)	20 (21.74)		
**Acute endometritis**				0.103	0.748
No	40 (86.96)	41 (89.13)	81 (88.04)		
Yes	6 (13.04)	5 (10.87)	11 (11.96)		
**Neonatal asphyxia**				0.000	1.000
No	44 (95.65)	45 (97.83)	89 (96.74)		
Yes	2 (4.35)	1 (2.17)	3 (3.26)		
**Neonatal pneumonia**				0.000	1.000
No	44 (95.65)	44 (95.65)	88 (95.65)		
Yes	2 (4.35)		4 (4.35)		

NVD: normal vaginal delivery. AVD: assisted vaginal delivery. CS: cesarean section.

* p<0.05 significance level.

## DISCUSSION

This study highlights the potential positive impact of labor dance on labor induction, anxiety levels in expectant mothers, and maternal-fetal outcomes within the context of our setting. Our findings suggest that labor dance may be an effective adjunct intervention that enhances the efficacy of labor induction, leading to a reduction in both the IOL and the first stage of labor in pregnant women with TPROM. Additionally, labor dance within our sample appeared to lower the incidence of chorioamnionitis and alleviate anxiety during labor induction. Furthermore, within our study population, it may increase the success rate of vaginal delivery.

Our findings are particularly noteworthy as they reveal the potential of labor dance as an adjunctive intervention that improves labor induction efficiency and shortens labor duration. This aligns with the concept that labor dance provides a unique form of physical activity that promotes relaxation among pregnant women. It may also influence the release of endogenous hormones, such as oxytocin, which can enhance uterine contractions and cervical dilation, thus initiating labor. Additionally, labor dance may modify anxiety responses in the brain’s limbic system, a key area associated with emotional regulation. Some studies have shown that dancing to music during labor can reduce anxiety in pregnant women^11,22^. The vertical motion involved in dance can also promote the secretion of endogenous oxytocin by improving the alignment of the fetal head with the cervix, thereby enhancing the effectiveness of IOL^23^. Moreover, labor dance may serve as a distraction, effectively reducing stress during oxytocin-induced labor^24^. Our study also revealed that labor dance could shorten the duration of the first stage of labor, consistent with the findings of Zhang et al.^8^. However, we did not observe a significant impact of labor dance on the total labor duration, possibly due to the limited scope of our intervention, which focused primarily on the induction process.

Pregnant women with TPROM may be particularly prone to anxiety when labor is induced with oxytocin. A substantial body of evidence suggests that anxiety in these women stems from the perception of an unpredictable and uncontrollable situation and the stress associated with uterine contractions^25,26^. Our findings indicate that labor dance effectively reduces anxiety during contraction induction, consistent with studies by McCaffrey et al.^27^ and Akin et al.^28^ who found that music and dance provide physical and emotional support to pregnant women, thereby alleviating anxiety. However, although T1 was assessed at the same clinical endpoint for all participants, the varying interval from T0 to T1 should be taken into account when interpreting between-group differences in anxiety. Further research is needed to examine the effects of dance- and music-based interventions on mood and physiological responses during labor induction, as well as the mechanisms underlying these effects.

Although previous studies have shown that labor dance positively influences childbirth^8,29^, few have examined its effect on postpartum bleeding. Our data revealed no association between labor dance and the volume of postpartum bleeding. In our study, the intervention occurred from the onset of oxytocin IOL to the latent phase of labor, whereas previous studies applied labor dance during the first stage of labor^10,11^.

We also examined the relationship between labor dance and mode of delivery. Although no statistically significant between-group difference was observed in the rate of normal vaginal delivery, the intervention group showed a numerically higher proportion of vaginal births. This finding should be interpreted cautiously, but it may suggest a potential supportive role of labor dance in facilitating labor progress. Labor dance may therefore facilitate vaginal delivery by promoting fetal descent and enhancing maternal–fetal coordination – both well-established determinants of the head-to-cervix force^30^. Similar mechanisms have been proposed in previous studies and reviews of mobility, and position-based labor support interventions, which suggest that maternal mobility may support labor progress and maternal comfort^9,31^. However, given the lack of statistical significance in delivery mode outcomes and the limited sample size of the present study, no definitive conclusion can be drawn. Larger studies are needed to clarify whether labor dance has a meaningful effect on the mode of delivery.

Our study also noted a significant reduction in the incidence of chorioamnionitis in women with TPROM during IOL. This positive effect is consistent with the study by Triebwasser et al.^32^, which aims to shorten the induction and reduce the risk of infection. However, due to limitations in statistical power in our study, this finding must be interpreted with caution.

Conversely, no significant differences were observed in the incidence of acute endometritis, neonatal asphyxia, or pneumonia between the intervention and control groups. This consistency may be attributed to the standardized medical care provided to both groups, including timely antibiotic administration, physical cooling, oxygen therapy, and emotional support, all of which ensured the safety of both mothers and infants. These findings align with prior research emphasizing the importance of prompt intervention and comprehensive management of complications during childbirth to minimize infections^33^.

### Strengths and limitations

Our study, as an RCT, has a robust study design, with findings that have several implications for clinical practice and research. However, several limitations should be acknowledged. First, because some participants did not complete the assigned intervention, the primary analysis was conducted on a per-protocol population rather than a strict intention-to-treat population. Although exclusions were numerically balanced between groups and were mostly attributable to clinical or personal circumstances, the possibility of attrition-related selection bias cannot be fully excluded. Therefore, the findings are most applicable to women who are able to complete the labor dance intervention as intended. Second, the eligibility criteria were restrictive. Only low-risk, healthy primiparous women aged 18–35 years with a modified Bishop score ≥7 were enrolled. Although this design reduced clinical heterogeneity and minimized confounding related to cervical ripening, it also limited generalizability. Because a modified Bishop score ≥7 indicates a favorable cervix suitable for direct oxytocin induction without prior cervical ripening, the findings are most relevant to women with TPROM who have favorable cervical conditions at baseline. Their applicability to women with less favorable cervical status remains uncertain. Third, walking was routinely encouraged in both groups as part of standard obstetric care. Although this did not constitute a structured or protocolized co-intervention, it may have reduced the contrast between groups and attenuated the observed intervention effect. Fourth, because of the behavioral and nonpharmacological nature of the intervention, blinding of participants and intervention providers was not feasible. Although outcome assessors and statisticians were blinded to group allocation, subjective outcomes such as maternal anxiety may still have been influenced by performance or detection bias. Finally, this was a single-center study with a relatively small sample size, which limited the precision of estimates for less frequent outcomes, particularly chorioamnionitis. Therefore, the observed difference in infectious outcomes should be interpreted cautiously. Larger, multicenter, adequately powered randomized controlled trials with broader eligibility criteria, objective outcome measures, and longer follow-up are needed to confirm and extend these findings.

## CONCLUSIONS

This study provides preliminary evidence regarding the adjunctive use of labor dance during oxytocin-induced labor in primigravida women with TPROM. The findings suggest that labor dance may potentially offer potential benefits, including a shorter induction-to-delivery interval, a shorter first stage of labor among women who achieved vaginal delivery, and lower maternal anxiety scores. As a single-center exploratory trial, this study has important methodological limitations, including the use of per-protocol analysis and restricted eligibility criteria. Accordingly, the findings cannot be readily generalized to broader obstetric populations, particularly women with comorbidities, multiparous women, or those with less favorable cervical conditions. Larger, multicenter, adequately powered randomized controlled trials are needed to confirm these findings. In addition, because the present study did not assess long-term maternal or neonatal outcomes, further research with extended follow-up is warranted to evaluate the durability and safety of labor dance in this clinical context.

## Supplementary Material



## Data Availability

The data supporting this research are available from the authors on reasonable request.
